# Literary Notes

**Published:** 1906-01

**Authors:** 


### LITERARY NOTES.

THE "Bloodless Phlebolomist" in its January issue published the following papers:

"Appendicitis. As An Infective Inflammation" by Professor Robert T. Morris, A.M., M.D., of New York; "The Early Diagnosis of Pulmonary Tuberculosis" by H. Edwin Lewis, M.D., of New York; "Phagedenic Ulcer" by J. Bonnefin, M.R.C.S., of Leytonstone, England.

THE New United States Pharmacopeia makes many changes in the strength of drugs and preparations, reducing some, increasing others as much as double. The law recognises the current U. S. Pharmacopeia as the standard. To avoid accidents and damage suits on the one hand, and puzzling lack of results on the other, both the druggist and doctor must follow the same standard. As a convenient pocket reminder of these changes, the importance of which must be at once obvious to every physician and pharmacist, Messrs. Lea Brothers & Co., the Medical Publishers, of 706-10 Sansom Street, Philadelphia, and 111 Fifth Avenue New York, have issued for free distribution a carefully prepared leaflet giving an alphabetical list of the important changes. The strength of the preparation listed is given as in both the old and new U. S. P.

To aid in preventing untoward or negative results in the use of powerful drugs, this leaflet will prove handy and valuable. A postal card request will bring a copy to any physician, druggist, student or nurse.

---

W. B. Saunders Company, medical publishers, Philadelphia, announce that during January Fowler's Surgery will be ready. It is an entirely new work, presenting the science and art of surgery as practised today. It will consist of two octavo volumes of 725 pages each and containing 888 original illustrations. It is written by Professor George Ryerson Fowler, M.D., Brooklyn's distinguished surgeon, who is examiner in surgery on the State Board of Medical Examiners.

---

Messrs. Lea Brothers & Co. published early in January, 1906, a complete new work on dietics adapted to the use of practitioners and students of Medicine, nurses and the laity, entitled Food in Health and Disease.—By Robert F. Williams, M.A., M.D., professor of principles and practice of medicine in the Medical College of Virginia, Richmond. This book will be reviewed at an early date.

---

"A Trip to the Land of the Midnight Sun" is the title of a pretty little brochure of eighty-seven pages, written by Dr. Flavel B. Tiffany, of Kansas City, Mo. It is an account of a two months' vacation tour, during which Dr. Tiffany, accompanied by his wife, visited the north cape and other Scandanavian points of interest to the tourist. The story is told in easy, conversational fashion, is handsomely printed and profusely illustrated, and makes an entertaining half-hour's reading.

